# Assessing Excessive Keratinization in Acral Areas through Dermatoscopy with Cross-Polarization and Parallel-Polarization: A Dermatoscopic Keratinization Scale

**DOI:** 10.3390/jcm12227077

**Published:** 2023-11-14

**Authors:** Jacek Calik, Bogusław Pilarski, Monika Migdał, Natalia Sauer

**Affiliations:** 1 Old Town Clinic, 50-043 Wroclaw, Poland; monika.migdal@oldtownclinic.pl; 2 Department of Clinical Oncology, Wroclaw Medical University, 50-556 Wrocław, Poland; 3 Cerko Sp. z o.o. Sp.k, Al. Zwycięstwa 96/98, 81-451 Gdynia, Poland; bpilarski@cerko.pl; 4 Faculty of Pharmacy, Wroclaw Medical University, 50-556 Wrocław, Poland

**Keywords:** excessive keratinization, dermatoscopy, dermatoscopic keratinization scale

## Abstract

Excessive epidermal hyperkeratosis in acral areas is a common occurrence in dermatology practice, with a notable prevalence of approximately 65% in the elderly, especially in plantar lesions. Hyperkeratosis, characterized by thickening of the stratum corneum, can have various causes, including chronic physical or chemical factors, genetic predispositions, immunological disorders, and pharmaceutical compounds. This condition can significantly impact mobility, increase the risk of falls, and reduce the overall quality of life, particularly in older individuals. Management often involves creams containing urea to soften hyperkeratotic areas. Currently, subjective visual evaluation is the gold standard for assessing hyperkeratosis severity, lacking precision and consistency. Therefore, our research group proposes a novel 6-point keratinization scale based on dermatoscopy with cross-polarization and parallel-polarization techniques. This scale provides a structured framework for objective assessment, aiding in treatment selection, duration determination, and monitoring disease progression. Its clinical utility extends to various dermatological conditions involving hyperkeratosis, making it a valuable tool in dermatology practice. This standardized approach enhances communication among healthcare professionals, ultimately improving patient care and research comparability in dermatology.

## 1. Introduction

Excessive epidermal hyperkeratosis in acral areas represents a frequently encountered phenomenon within the realm of routine clinical practice in dermatology. Notably, it exhibits a heightened prevalence, approaching approximately 65%, among the geriatric demographic, particularly in the context of plantar lesions [1]. It is associated with female gender, hallux valgus, toe deformity, increased ankle flexibility, and time spent on feet during the day, but is not associated with obesity, limb dominance, forefoot pain, or foot posture. Although there is a wide range of lesion distribution patterns, most can be classified into medial, central, or lateral groups [2]. Hyperkeratosis, characterized by the thickening of the stratum corneum, is classified as either orthokeratotic, where keratinocyte maturation is preserved, or parakeratotic, where nuclei retention indicates delayed maturation [3]. It can be linked to dyskeratosis, representing premature or abnormal keratinization of individual keratinocytes, often aiding in histological diagnosis alongside other skin biopsy abnormalities. Epidermal hypertrophy, a benign skin alteration, manifests with acanthosis and hyperkeratosis, indicating increased thickness of the keratinocyte layers. Hyperkeratosis, characterized by an abundance of keratinized tissue, is predominantly an adaptive defensive response of the epidermis to chronic cutaneous trauma [3,4]. Excessive epidermal hyperkeratosis can be precipitated by various factors, including chronic physical or chemical insults (such as prolonged friction or the application of aggressive soaps), genetic predispositions, or underlying immunological disorders [5,6]. Furthermore, hyperkeratosis can develop due to various pharmaceutical compounds primarily administered in oncology treatments [7]. A hyperkeratotic drug reaction is frequently observed with medications like tyrosine kinase inhibitors, cytotoxic chemotherapy drugs, as well as immunomodulators or immune checkpoint inhibitors [8,9,10,11,12].

In the geriatric population, where plantar hyperkeratotic lesions are common, they are associated with pain, mobility impairment, and functional limitations. Plantar lesions often induce discomfort, reduced gait velocity, compromised postural stability, and challenges in negotiating stairs, consequently leading to functional impairment and decreased autonomy among elderly individuals [13,14]. An indicator of the prevalence and ramifications of hyperkeratotic lesions within the community for the podiatric workforce is that the debridement of such lesions constitutes a substantial portion, up to 75%, of the podiatrist’s caseload [14]. Remarkably, 84% of individuals seeking intervention for hyperkeratotic lesions opt to consult with a podiatrist [2]. Hyperkeratosis is a consequence of aberrant mechanical forces exerted on the skin, thereby inciting an augmented keratinization process. This, in turn, triggers an accelerated proliferation of epidermal cells and a reduced rate of desquamation, culminating in the hypertrophy of the stratum corneum [15]. The augmented stratum corneum thickness amplifies the area over which mechanical forces can be dissipated. This inherent process of asymptomatic hyperkeratosis (physiological hyperkeratosis) plays a pivotal role in shielding the skin and underlying soft tissues from mechanical trauma. Nonetheless, hyperkeratosis assumes a pathological character when the buildup of keratinized material reaches a point where it precipitates tissue injury and discomfort, possibly via the release of inflammatory mediators or as a consequence of the central keratin plug’s pressure on subjacent nerves [16,17].

This condition can pose challenges to ambulation and elevate the susceptibility to falls, particularly in the geriatric population, thereby contributing to a reduction in their overall quality of life [18,19,20,21]. Hyperkeratosis may be managed through the application of creams containing urea, which facilitate the dissolution of the intercellular matrix within the stratum corneum’s cellular structure [22,23]. Empirical data suggests that optimal effectiveness, approximately 90%, is achieved when urea concentrations reach 10% [24]. Furthermore, the most pronounced and potentially expedited outcomes are observed at concentrations up to 40% [25]. In addition, magisterial prescriptions with silver nitrate and balsam of Peru can be effective in treating cracks in pressure areas, erosions caused by the removal of scales, and hyperkeratotic areas [26]. This process promotes the shedding of scaly skin, ultimately leading to the softening of hyperkeratotic areas. The mitigation of the lesion(s), utilization of suitable padding, protective measures, orthotic devices designed for accommodating deformities, insole applications, moisturizing agents, and gentle keratolytic agents administered at routine treatment intervals prove to be efficacious in preserving the well-being and facilitating the functionality of the foot [27].

Currently, the gold standard for assessing the severity of keratosis relies on visual evaluation, a subjective approach that employs descriptive terms like mild, moderate, severe, or very severe [28,29,30,31,32,33,34]. Regrettably, these terms often fail to convey a precise clinical picture to the recipient. Furthermore, when hyperkeratosis accompanies various dermatological conditions, these descriptions can significantly vary from each other, posing a challenge in providing accurate literature reviews of clinical cases. Consequently, this study aims to demonstrate the utility of dermatoscopy in meticulously evaluating the extent of hyperkeratosis in acral areas. This innovative approach facilitates a quantitative classification of hyperkeratosis severity, ensuring an objective assessment of treatment outcomes. A comprehensive evaluation of keratosis severity is instrumental in delineating a patient’s therapeutic requirements. It aids in the precise selection of topical preparations, treatment duration determination, and keratolytic agent application frequency. This study endeavors to establish a practical and expeditious assessment scale for excessive skin keratosis in acral regions. The scale is devised based on manual dermatoscopic evaluations employing both cross-polarized and parallel-polarized illumination techniques.

## 2. Materials and Methods

### 2.1. Patients Group

Observations conducted from September 2018 to June 2023 with the aim of establishing a scale for keratinization were executed on patients who had undergone dermoscopic examinations at the Old Town Clinic in Wrocław, Poland. These examinations were conducted using FotoFinder devices equipped with Medicam 800, Medicam 1000, and Medicam 1000S cameras (FotoFinder Systems GmbH, Bad Birnbach, Germany). Participants in this study were selected and qualified by dermatologists and oncologists. Study participants were exclusively recruited from healthy individuals who had not previously undergone localized therapeutic interventions within the regions under investigation. Patients with psoriasis, keratoderma, tinea pedis, and similar conditions were not included in the study. In total, 5434 individuals, spanning from 2 weeks to 98 years of age, were subjected to scrutiny, with females constituting 61% of the cohort. The study focused on the assessment of keratinization in the acral regions, particularly the hands and feet. Furthermore, it was discerned that, within the same patient, variances in the degree of keratinization were evident across distinct acral regions.

### 2.2. Dermoscopic Evaluation of Keratinization

Non-immersion dermatoscopy with cross-polarized and parallel-polarized light sources was employed to assess excessive keratinization. Cross-polarization was achieved through the utilization of the Foto Finder device equipped with the Medicam 1000 camera (FotoFinder Systems GmbH, Bad Birnbach, Germany), as well as the handheld Derm Lite DL-5 dermatoscope (DermLite LLC, San Juan Capistrano, CA, USA). The introduction of any immersion fluid in dermatoscopy would disrupt the quantitative evaluation of epidermal keratin, which is perceptible as white structures, including white lines and white areas.

**Figure 1:**
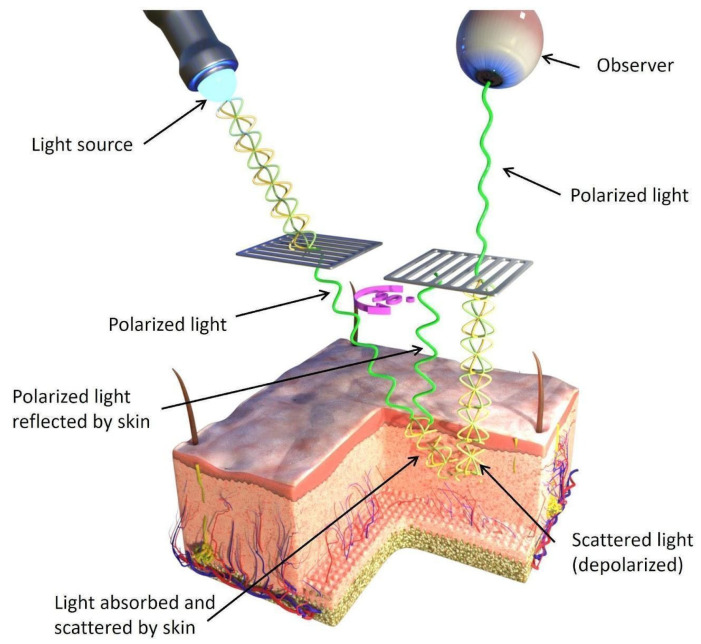
Passage of reflected light waves from deeper layers of the skin through a polarizing plate to the observer (human eye) in cross-polarized dermatoscopy.

Dermoscopy employing cross-polarized light, wherein the polarizing plates are oriented at a 90° angle, presently stands as the gold standard in dermatoscopic diagnostics for the majority of cutaneous lesions. The presence of polarized light enables the comprehensive assessment of both superficial and deep structures, all without necessitating the use of an immersion medium. Figure 1 schematically depicts cross-polarization dermatoscopy.

**Figure 2:**
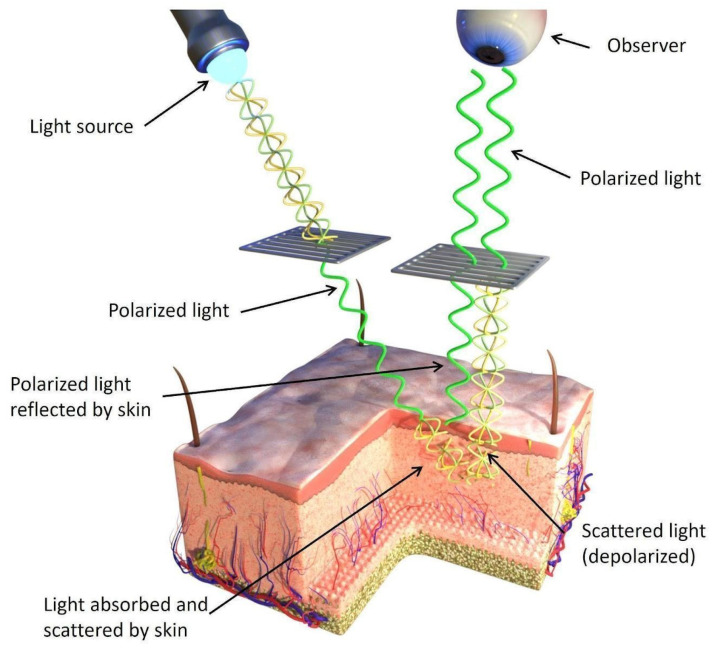
Passage of reflected light waves from deeper layers of the skin through a polarizing plate to the observer (human eye) in parallel-polarized dermatoscopy.

Parallel-polarized dermatoscopy (where the polarizing plates in the device are aligned parallel) potentially offers superior imaging capabilities for lesions located exclusively within the epidermis. Examination using parallel polarization is depicted in Figure 2.

Recently, a new DermLite dermatoscope with the added functionality of assessing skin lesions using both cross-polarization and parallel-polarization has entered the market. In this study, the authors employed these dermatoscopic evaluation techniques to identify characteristic features, which were then utilized to construct a 6-point scale for keratinization in acral areas.

Cross-polarization and parallel-polarization techniques are fundamental concepts in the manipulation of polarized light [28]. These techniques are rooted in the properties of light waves and their interaction with polarizing elements.

In the case of polarized light, light, which is an electromagnetic wave, can vibrate in multiple directions. Nonpolarized light consists of waves vibrating in various planes, while polarized light vibrates in a single plane. This property is known as polarization. Cross-polarization involves using two polarizers with perpendicular orientations concerning the incident and reflected light. This arrangement effectively eliminates glare and specular highlights. It is particularly valuable for examining birefringent structures that might otherwise remain invisible. In contrast, parallel-polarization occurs when both polarizers are aligned in the same orientation. This technique is instrumental for visualizing surface and subsurface elements, allowing for enhanced detail in certain applications. These polarization techniques find applications in various fields, including dermatology. In dermatoscopy, cross-polarization enhances the visualization of deeper skin structures, while parallel-polarization aids in the examination of surface features.

## 3. Results

A 6-point scale for keratinization in acral areas.

In the context of quantifying keratinization within the acral regions, an intricate 6-point grading system has been devised to meticulously categorize and evaluate the diverse manifestations of hyperkeratosis (Table 1). This scale is delineated as follows:

Grade I

Lack of hyperkeratosis: The absence of white structures is noted in both furrows and ridges. Small foci of white scale unrelated to furrows may be encountered.

Grade II

Minimal hyperkeratosis: Interrupted white lines are observed within furrows, corresponding to focal keratinization in the furrows. Ridges are devoid of white structures.

Grade III

Moderate hyperkeratosis: Delicate, thin white lines are observed within furrows. These white lines correspond to keratin masses located within the furrows.

Grade IV

Severe hyperkeratosis: Thick white lines are observed within furrows. These white lines correspond to keratin masses filling nearly the entire furrow and occasionally extending beyond their boundaries, giving the impression of jagged edges.

Grade V

Intensely severe hyperkeratosis: Thick white lines are interconnected by white bridges. Epidermal fissures, typically oriented perpendicular to furrows and ridges, may manifest as two thick, parallel white lines.

Grade VI

In addition to the very thick interconnected white lines, formless yellow areas are observed, corresponding to non-structured keratin masses that have lost the typical furrowing seen in acral areas. Clinically, these changes appear cohesive, and in the vicinity of the yellow keratinized areas, epidermal fissures occur.

**Table 1:** Dermoscopic views corresponding to the grades of keratosis.

Grade	Dermoscopic View
Grade I	Foto Finder dermatoscopy, cross-polarization
	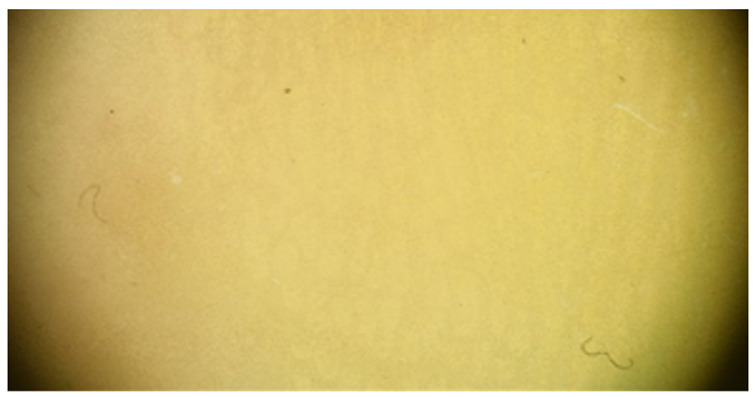
	DL-5 dermatoscopy, cross-polarization
	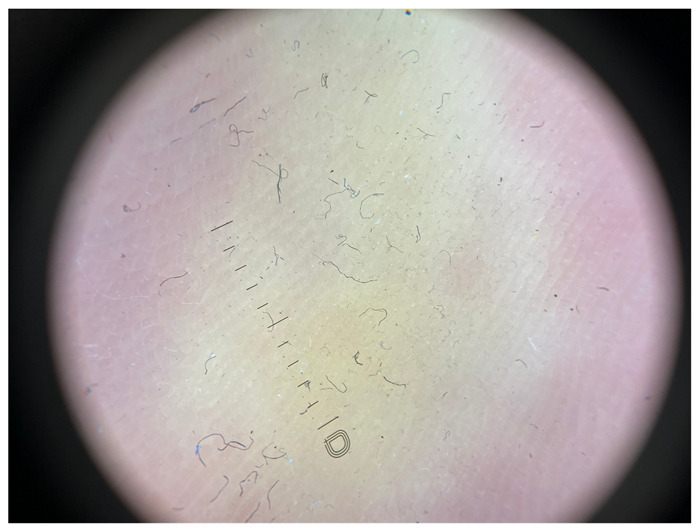
	DL-5 dermatoscopy, parallel polarization
	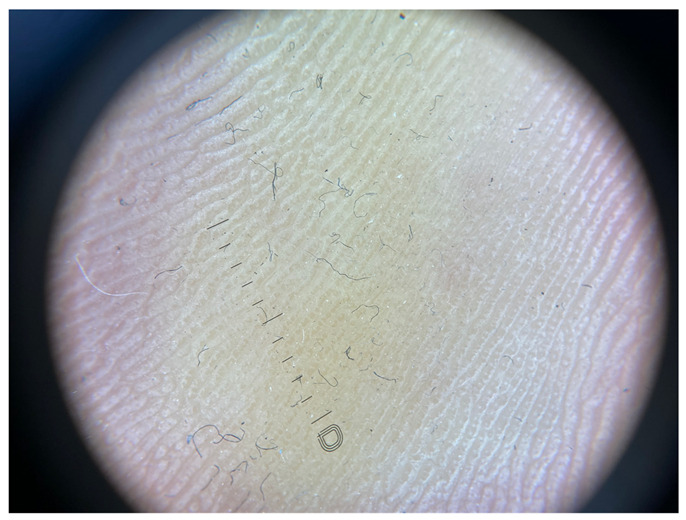
Grade II	Foto Finder dermatoscopy, cross-polarization
	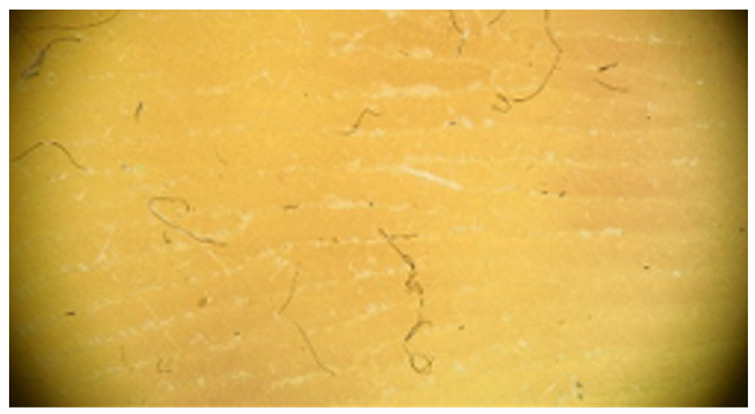
	DL-5 dermatoscopy, cross-polarization
	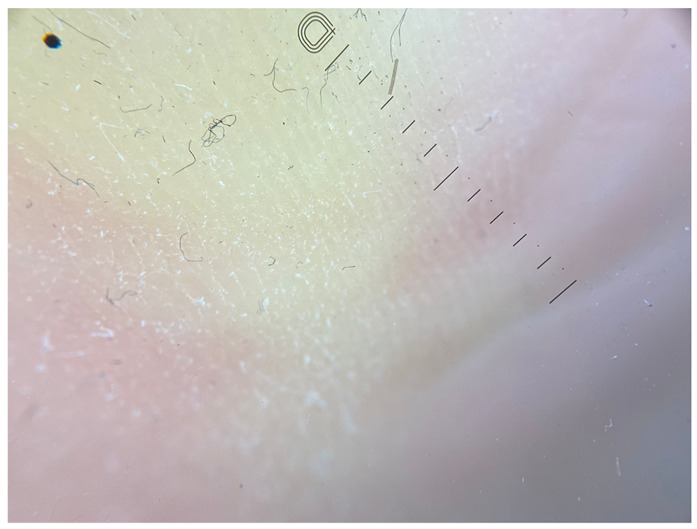
	DL-5 dermatoscopy, parallel polarization
	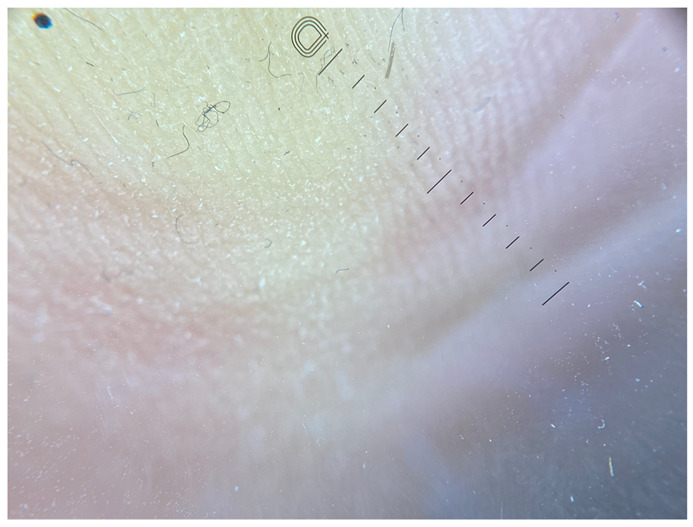
Grade III	Foto Finder dermatoscopy, cross-polarization
	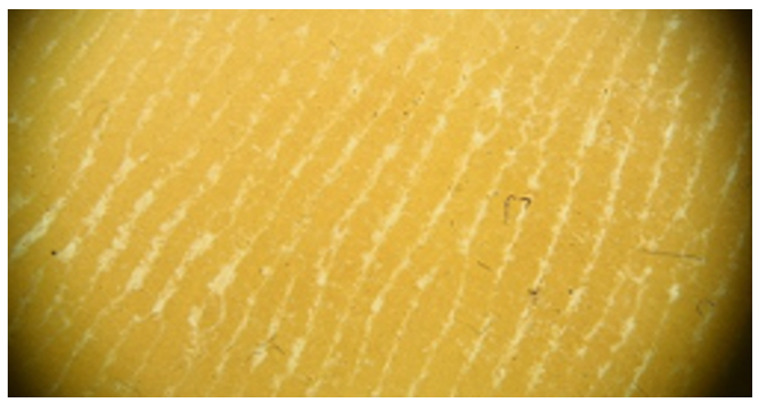
	DL-5 dermatoscopy, cross-polarization
	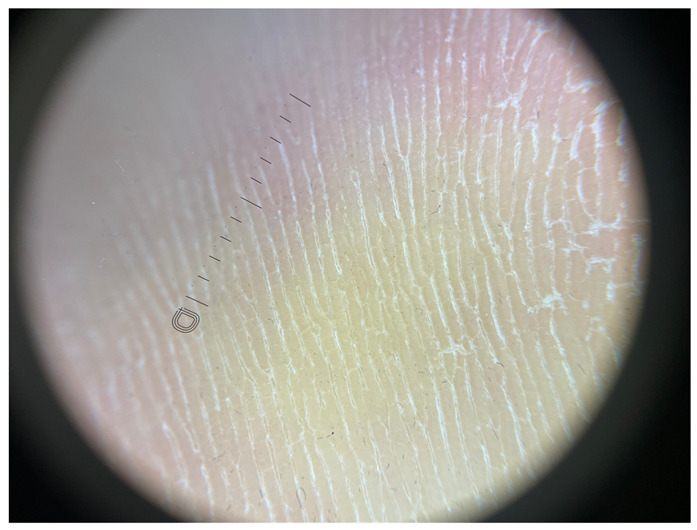
	DL-5 dermatoscopy, parallel polarization
	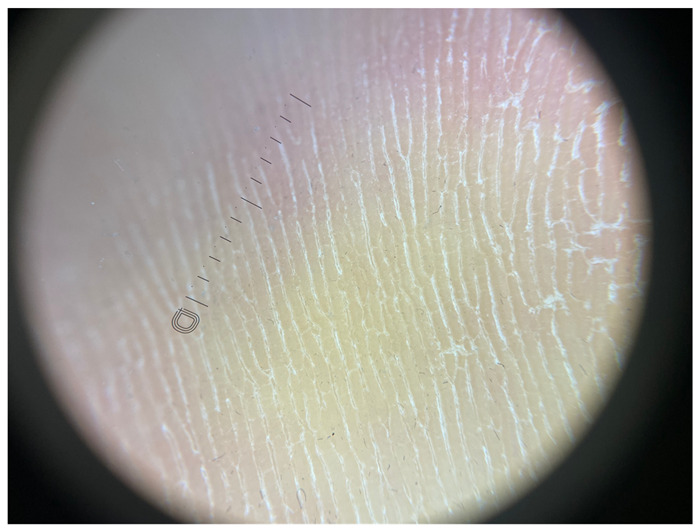
Grade IV	Foto Finder dermatoscopy, cross-polarization
	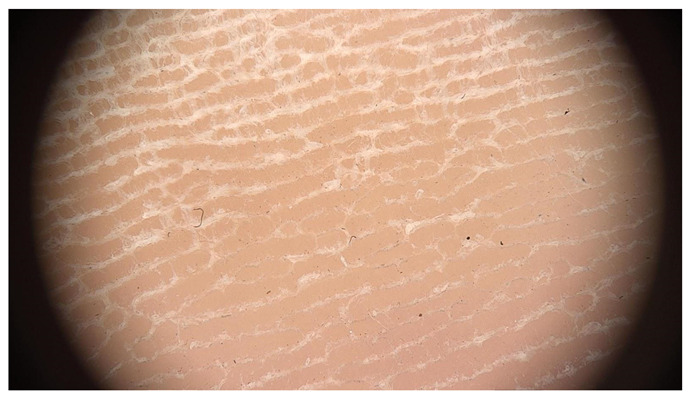
	DL-5 dermatoscopy, cross-polarization
	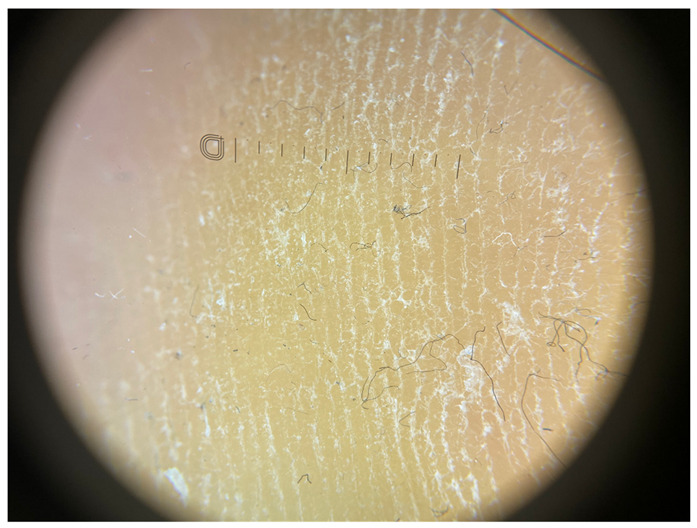
	DL-5 dermatoscopy, parallel polarization
	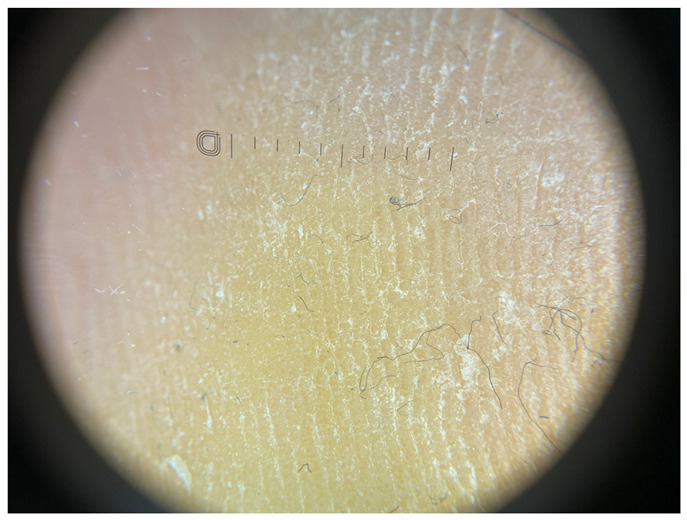
Grade V	Foto Finder dermatoscopy, cross-polarization
	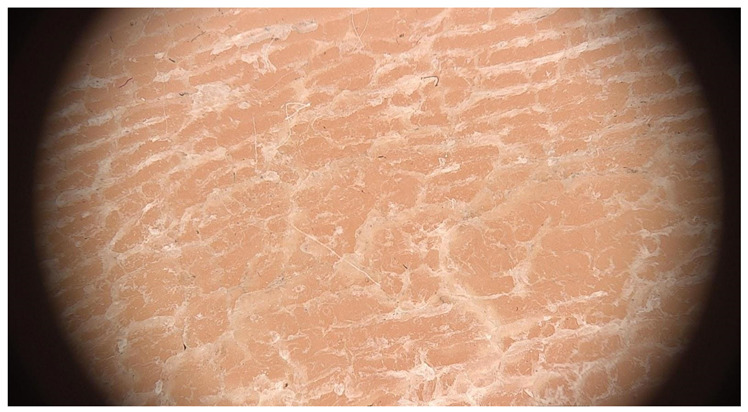
	DL-5 dermatoscopy, cross-polarization
	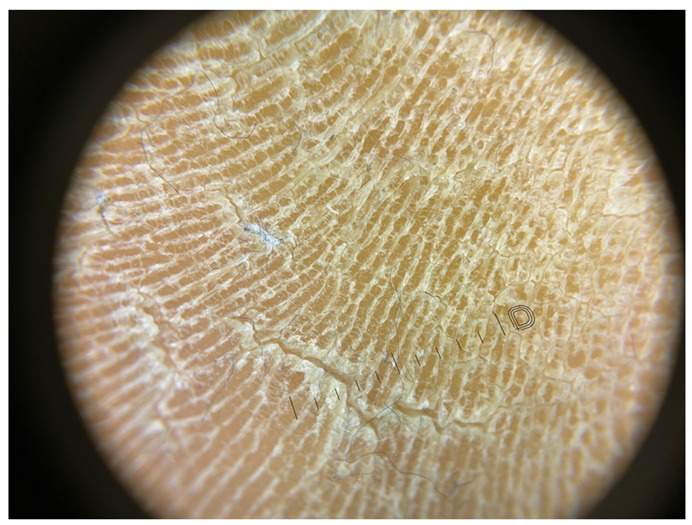
	DL-5 dermatoscopy, parallel polarization
	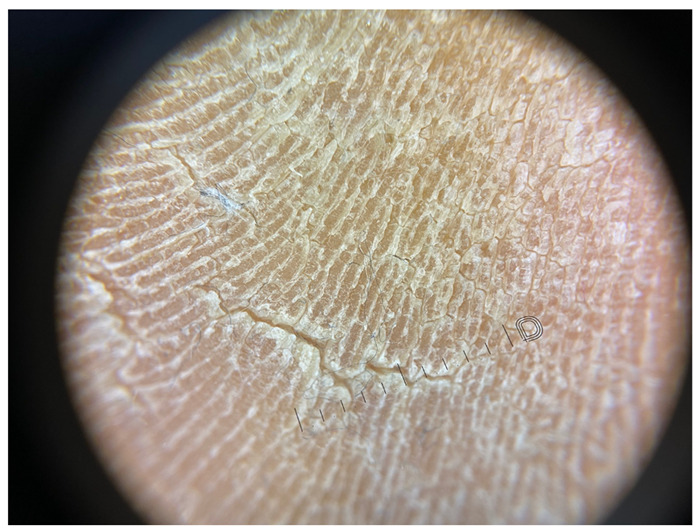
Grade VI	Foto Finder dermatoscopy, cross-polarization
	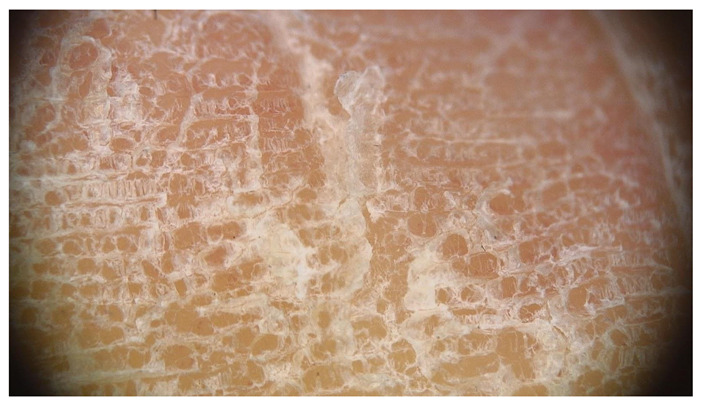
	DL-5 dermatoscopy, cross-polarization
	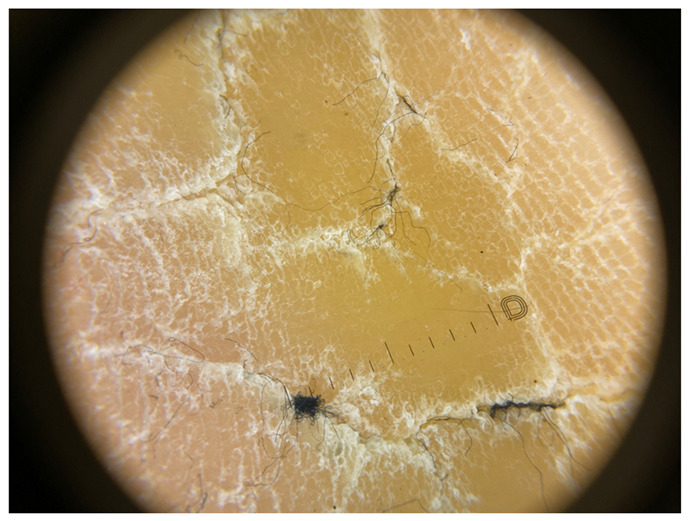
	DL-5 dermatoscopy, parallel polarization
	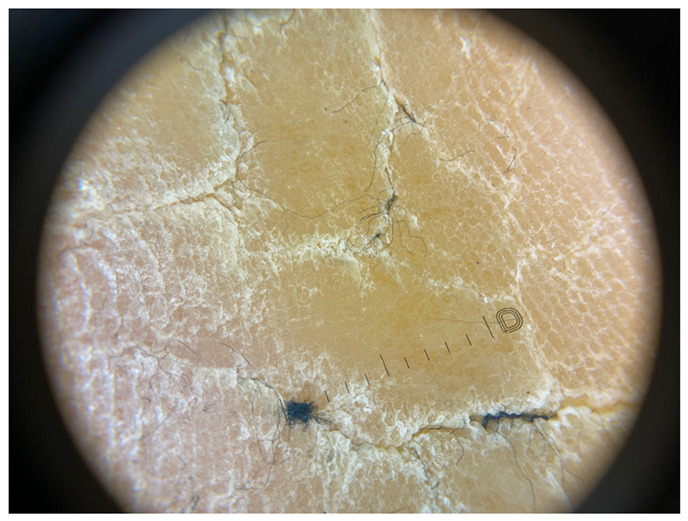

**Figure 3:**
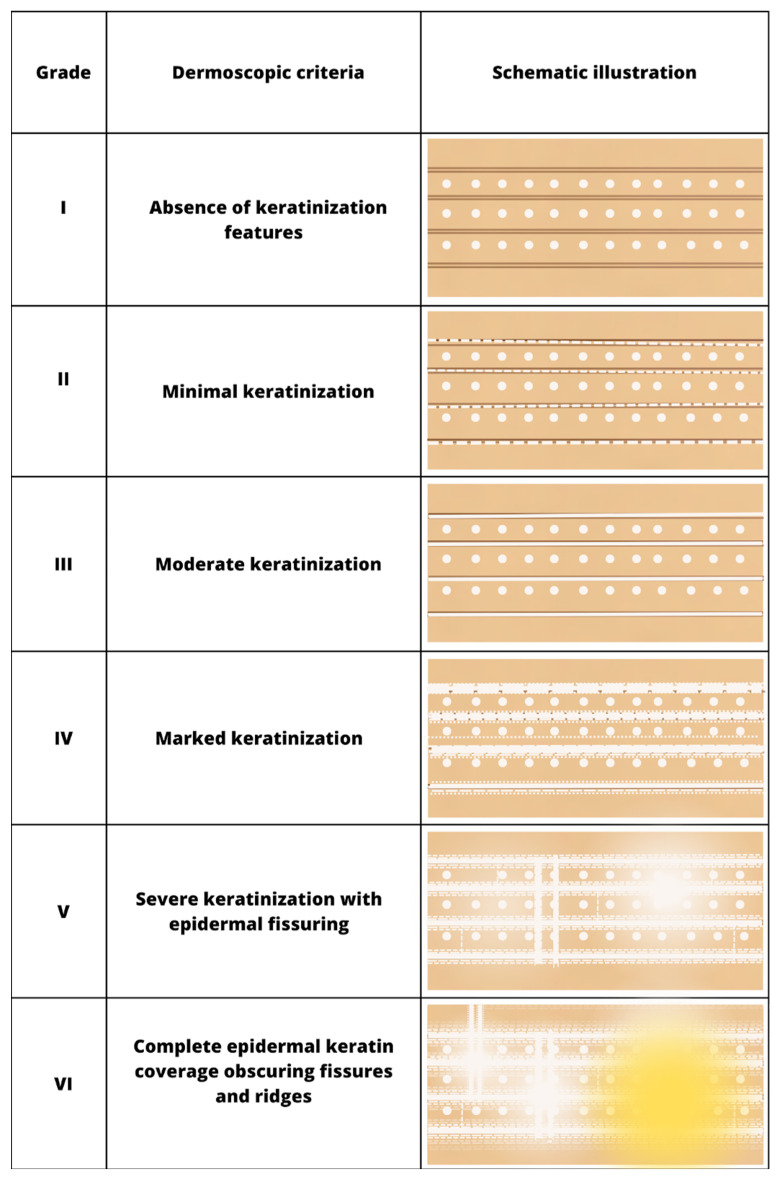
Schematic representation of dermatoscopic 6-point keratosis scales. Grade I: A lack of hyperkeratosis, with no white structures observed in the furrows and ridges. Small foci of white scale may be found independently of furrows.; Grade II: Interrupted white lines within furrows represent minimal hyperkeratosis, corresponding to focal keratinization. Ridges remain devoid of white structures.; Grade III: Thick white lines within furrows signify moderate hyperkeratosis, indicating keratin masses situated within the furrows.; Grade IV: Thick white lines within furrows denote severe hyperkeratosis, with keratin masses filling almost the entire furrow and occasionally extending beyond, creating jagged edges.; Grade V: Thick white lines interconnected by white bridges depict intensely severe hyperkeratosis, often accompanied by epidermal fissures oriented perpendicular to furrows and ridges.; Grade VI: Besides thick interconnected white lines, the presence of homogeneous yellow areas signifies non-structured keratin masses that have lost typical furrowing seen in acral areas. Clinically, these changes appear cohesive, with epidermal fissures occurring near the yellow keratinized areas.

After conducting a rigorous and comprehensive analysis of the dermatoscopic depictions that illustrate the intricacies of the six-point keratinization scale, we are now prepared to delve into a more concise and visually informative representation of these criteria. This graphical exposition, presented in Figure 3, serves as a schematic rendering that facilitates a deeper comprehension of the dermatological assessment at hand. Figure 3 offers a condensed yet highly illustrative visual guide that delineates the key dermatoscopic criteria associated with each grade of keratosis. This schematic representation allows for a more accessible and systematic understanding of the varying characteristics and features that underpin the assessment of keratinization in dermatology.

## 4. Discussion

Epithelial tissues serve to safeguard the underlying structures from external factors, including physical harm, infections, drying out, UV radiation, and heat loss and also play a role in preserving the body’s internal balance (homeostasis) [35]. Keratinization, also known as cornification, is a process of cellular differentiation that keratinocytes experience as they transition from their initial, actively dividing state in the stratum basale to ultimately become fully differentiated, toughened cells filled with protein. These cells make up a structurally and functionally unique surface layer characterized by the presence of keratin, such as the stratum corneum [36]. Keratin proteins exhibit a consistent distribution pattern throughout the diverse layers of epithelial tissues, providing valuable insights into pathological processes [35]. This uniform distribution is indicative of underlying disease processes. Additionally, numerous disorders have been linked to abnormalities in keratins and their associated proteins, which can manifest in the skin, oral mucosa, or both. These conditions often result in a wide range of clinical manifestations, making the study of keratin-related disorders a significant area of research in dermatology and oral medicine.

The assessment of excessive keratinization in acral areas is of clinical importance, as it can aid in the diagnosis and management of various dermatological conditions [35]. Dermatoscopy, which involves the use of polarized light sources, has become a valuable tool in evaluating skin lesions and conditions. Systematizing the severity of hyperkeratosis in acral areas appears to hold practical utility in daily clinical practice [37].

This study introduces a novel approach to assess keratinization in acral areas through dermatoscopy with both cross-polarization and parallel-polarization techniques, resulting in the development of a 6-point keratinization scale. The scale proposed by the authors, ranging from Grade I to Grade VI, provides a clear and structured framework for assessing keratinization. Each grade is characterized by specific dermatoscopic features, including the presence or absence of white structures, the thickness of white lines, and the presence of yellow keratinized areas. The use of dermatoscopic images for each grade further enhances the understanding of these features, making it easier for clinicians to apply the scale in their practice. Similar scales, such as the Psoriasis Area and Severity Index (PASI) for assessing psoriasis severity and the Scoring Atopic Dermatitis (SCORAD) scale for evaluating the extent and severity of atopic dermatitis, have already demonstrated their effectiveness in clinical practice. These scales have significantly enhanced the quality of clinical assessment, making them commonplace tools in medical practice [38,39].

This classification can be employed to determine the duration and intensity of therapy while also enabling precise assessment of treatment response. The dermatoscopic image under cross-polarization appears sharper and more readily interpretable, although it may not fully capture the issue when examining photographs taken in parallel polarization. Parallel polarization reveals more surface details, allowing for a more precise evaluation of keratosis severity. The use of cross-polarization facilitates easier classification of changes into specific grades according to the scale presented above. However, we suggest that employing the more commonly used cross-polarization in the routine assessment of acral keratinization would be a better and more straightforward approach to facilitate communication among medical professionals.

The ability to assess keratinization in the acral areas using this scale has several clinical implications. Firstly, it can aid in the early detection and differentiation of various dermatological conditions that involve hyperkeratosis, such as palmoplantar keratoderma or psoriasis [40,41]. Its importance will be particularly pronounced in the evaluation of plantar hyperkeratotic patterns in older patients, as hyperkeratotic alterations in this demographic can result in gait disturbances, reduced quality of life, and pose potential risks, particularly among individuals with diabetes [15,42,43].

In addition, it is important to note that keratinization plays a fundamental role in maintaining the structural integrity of various epithelial tissues, and mutations in keratin genes have been associated with specific tissue fragility disorders [44]. In our study, the scale was developed based on extensive observations of thousands of patients, demonstrating the physiological characteristics of keratinization in the acral regions. Therefore, this study is valuable for assessing whether specific changes in keratinization are perceived as normal or deviate from the norm. By identifying specific features and degrees of keratinization, it becomes possible to provide more precise descriptions of clinical cases in research publications. Therefore, understanding the process of keratinization and having a reliable assessment scale is pivotal in identifying and managing a wide range of dermatological disorders.

Furthermore, the scale can serve as a valuable tool for monitoring disease progression and treatment response and evaluating the effectiveness of the medications employed. Thus, changes in the grade of keratinization over time can provide insights into the effectiveness of therapeutic interventions. Furthermore, the scale can contribute to a standardized approach to dermatoscopic evaluation in clinical and research settings. By using a consistent grading system, clinicians can communicate more effectively about the severity of keratinization, leading to better patient care and improved research comparability. The introduction of a new scale for keratinization assessment in acral areas represents a significant advancement in the field of dermatology.

However, beyond the scale itself, there are several aspects regarding its impact and significance that are worth considering. First and foremost, it is crucial to understand how the new scale can influence patients. Effective communication of diagnoses and prognoses is pivotal for patients, subsequently affecting their comprehension and trust in healthcare professionals. The scale can aid in conveying health information in a more lucid manner, potentially leading to an increased level of trust in medical practitioners. Furthermore, the new scale can influence how patients perceive their condition, aiding in their understanding and acceptance of their health status and motivating them to adhere to medical recommendations. Another significant aspect is the global context. The scale should be in line with international standards and practices in dermatology. Adapting it to various cultures and countries can enhance its utility and dissemination, which is paramount in the global realm of medicine. Lastly, there is potential for collaboration with other medical fields, such as pathology and immunology. Integrating knowledge and tools from various disciplines can contribute to a more comprehensive assessment of keratinization changes. Collaborating with pathologists can assist in identifying pathological tissue changes affecting keratinization while working with immunologists can provide insights into the connections between immunological reactions and skin alterations.

## 5. Conclusions

In conclusion, the 6-point keratinization scale for acral areas is of paramount importance in the field of dermatology due to its multifaceted significance. This novel approach to assessing keratinization, developed through dermatoscopy with cross-polarization and parallel-polarization techniques, has far-reaching implications. It provides a structured and objective framework for evaluating hyperkeratosis severity, enabling precise disease monitoring, tailored treatment interventions, and assessment of treatment response. This scale transcends the limitations of subjective, descriptive terms, offering a standardized approach that greatly enhances communication among healthcare professionals. Facilitating consistent and accurate assessments significantly elevates the quality of clinical care and enhances research comparability in the field of dermatology. However, the current gold standard for assessing hyperkeratosis severity relies on subjective visual evaluation, which lacks precision and consistency. This limitation necessitated the development of a more objective and precise assessment tool, leading to the creation of the 6-point keratinization scale. This scale addresses the specific dermatoscopic features associated with each grade of keratinization, offering a detailed and comprehensive evaluation. It aids in precise treatment selection, determination of treatment duration, and application frequency of keratolytic agents. Furthermore, this standardized approach enhances communication among healthcare professionals, ultimately improving patient care and research comparability in dermatology. Beyond its clinical application, the scale has broader implications. It can enhance patient-physician communication, leading to better patient understanding and trust. Its adaptability to various cultural contexts can make it a global standard in dermatology, facilitating international collaboration and research. Additionally, collaboration with other medical fields, such as pathology and immunology, can provide a more holistic understanding of keratinization changes, advancing our knowledge of skin disorders.

## Data Availability

No new data were created or analyzed in this study. Data sharing is not applicable to this article.

## References

[1] García C.A., Soler F.C. (2017). Plantar Hyperkeratotic Patterns in Older Patients. Int. J. Gerontol..

[2] Spink M.J., Menz H.B., Lord S.R. (2009). Distribution and correlates of plantar hyperkeratotic lesions in older people. J. Foot Ankle Res..

[3] Farci F., Mahabal G.D. (2023). Hyperkeratosis. Encyclopedia of Parasitology.

[4] Sánchez-Rodríguez R., Martínez-Quintana R., Martínez-Nova A., Martínez-Rico M., Pedrera-Zamorano J.D., Chicharro-Luna E. (2023). Correlation between the foot pressure index and the prevalence of plantar hyperkeratosis. J. Tissue Viability.

[5] Vastarella M., Fabbrocini G., Sibaud V. (2020). Hyperkeratotic Skin Adverse Events Induced by Anticancer Treatments: A Comprehensive Review. Drug Saf..

[6] Carr E.S., Brown S.C., Fiala K.H. (2017). Painful nipple hyperkeratosis secondary to vemurafenib. Dermatol. Ther..

[7] Deutsch A., Leboeuf N.R., Lacouture M.E., McLellan B.N. (2020). Dermatologic Adverse Events of Systemic Anticancer Therapies: Cytotoxic Chemotherapy, Targeted Therapy, and Immunotherapy. Am. Soc. Clin. Oncol. Educ. Book Am. Soc. Clin. Oncol. Annu. Meet..

[8] Macdonald J.B., Macdonald B., Golitz L.E., LoRusso P., Sekulic A. (2015). Cutaneous adverse effects of targeted therapies: Part I: Inhibitors of the cellular membrane. J. Am. Acad. Dermatol..

[9] Ellis S.R., Vierra A.T., Millsop J.W., Lacouture M.E., Kiuru M. (2020). Dermatologic toxicities to immune checkpoint inhibitor therapy: A review of histopathologic features. J. Am. Acad. Dermatol..

[10] Greco A., Safi D., Swami U., Ginader T., Milhem M., Zakharia Y. (2019). Efficacy and Adverse Events in Metastatic Melanoma Patients Treated with Combination BRAF Plus MEK Inhibitors Versus BRAF Inhibitors: A Systematic Review. Cancers.

[11] Robert C., Mateus C., Spatz A., Wechsler J., Escudier B. (2008). Dermatologic symptoms associated with the multikinase inhibitor sorafenib. J. Am. Acad. Dermatol..

[12] Huang V., Hepper D., Anadkat M., Cornelius L. (2012). Cutaneous toxic effects associated with vemurafenib and inhibition of the BRAF pathway. Arch. Dermatol..

[13] Menz H.B., Lord S.R. (2001). Foot pain impairs balance and functional ability in community-dwelling older people. J. Am. Podiatr. Med. Assoc..

[14] Benvenuti F., Ferrucci L., Guralnik J.M., Gangemi S., Baroni A. (1995). Foot pain and disability in older persons: An epidemiologic survey. J. Am. Geriatr. Soc..

[15] Murray H.J., Young M.J., Hollis S., Boulton A. (1996). The association between callus formation, high pressures and neuropathy in diabetic foot ulceration. Diabet. Med..

[16] Booth J., McInnes A. (1997). The aetiology and management of plantar callus formation. J. Wound Care.

[17] Singh D., Bentley G., Trevino S.G. (1996). Callosities, corns, and calluses. BMJ.

[18] Demir B., Erden I., Ucak H., Demir S., Cicek D., Ozturk S. (2016). Quality of life in patients with calluses. Int. J. Dermatol..

[19] Jalali M., Mojgani P., Saeedi H., Azadinia F., Niksolat M., Ghorbani F. (2021). The relationship between common foot problems with falls and quality of life in older people. Int. J. Older People Nurs..

[20] Song Z., Ou J., Shu L., Hu G., Wu S., Xu X., Chen Z. (2022). Fall Risk Assessment for the Elderly Based on Weak Foot Features of Wearable Plantar Pressure. IEEE Trans. Neural Syst. Rehabil. Eng..

[21] Niu J., Zheng Y., Liu H., Chen X., Ran L. (2019). Stumbling prediction based on plantar pressure distribution. Work.

[22] Piquero-Casals J., Morgado-Carrasco D., Granger C., Trullàs C., Jesús-Silva A., Krutmann J. (2021). Urea in Dermatology: A Review of its Emollient, Moisturizing, Keratolytic, Skin Barrier Enhancing and Antimicrobial Properties. Dermatol. Ther..

[23] Annunziata M.C., Cacciapuoti S., Cosentino C., Fabbrocini G. (2020). Urea-containing topical formulations. Int. J. Clin. Pract..

[24] Pan M., Heinecke G., Bernardo S., Ba M.P., Tsui C., Levitt J. (2013). Urea: A comprehensive review of the clinical literature. Dermatol. Online J..

[25] Nwabudike L.C., Tatu A.L. (2018). Magistral Prescription with Silver Nitrate and Peru Balsam in Difficult-to-Heal Diabetic Foot Ulcers. Am. J. Ther..

[26] Starace M., Alessandrini A., Piraccini B.M. (2020). Clinical evidences of urea at high concentration on skin and annexes. Int. J. Clin. Pract..

[27] George D.H. (1993). Management of hyperkeratotic lesions in the elderly patient. Clin. Podiatr. Med. Surg..

[28] Chaduvula J., Chintada D., Vijayashree J., Chalamalasetty S.S., Vudayana K., Vaggu A. (2023). A Clinico-Epidemiological Study of Hyperkeratotic Palmoplantar Lesions and Its Correlation with Dermoscopy and Histopathology in a Tertiary Care Center. Cureus.

[29] Shah V.H., Rambhia K.D., Mukhi J.I., Singh R.P., Kaswan P. (2022). Clinico-investigative Study of Facial Acanthosis Nigricans. Indian Dermatol. Online J..

[30] Emre S., Metin A., Demirseren D.D., Yorulmaz A., Onursever A., Kaftan B. (2010). Netherton sendromlu kardeş olgular. Turkish J. Med. Sci..

[31] Balbinotti R.R., Grossi F.S., Perez A.V., Sbaraini M., Chagas L.B., Tregnago A.C., Vettorazzi J. (2021). Nonablative radiofrequency in the treatment of refractory vulvar lichen sclerosus: A case series. JAAD Case Rep..

[32] Gorai S., Ahmad S., Raza S.S.M., Khan H.D., Raza M.A., Etaee F., Cockerell C.J., Apalla Z., Goldust M. (2022). Update of pathophysiology and treatment options of seborrheic keratosis. Dermatol. Ther..

[33] Sonthalia S., Pasquali P., Agrawal M., Sharma P., Jha A.K., Errichetti E., Lallas A., Sehgal V.N. (2019). Dermoscopy Update: Review of Its Extradiagnostic and Expanding Indications and Future Prospects. Dermatol. Pract. Concept..

[34] Acar A., Karaarslan I. (2022). Comparison of Actinic Keratosis and Severity Index with Physician Global Assessment and Total Lesion Count and the Ability to Predict Skin Cancer. Dermatol. Pract. Concept..

[35] Shetty S., Gokul S. (2012). Keratinization and its Disorders. Oman Med. J..

[36] Deo P.N., Deshmukh R. (2018). Pathophysiology of keratinization. J. Oral Maxillofac. Pathol..

[37] Ring C., Cox N., Lee J.B. (2021). Dermatoscopy. Clin. Dermatol..

[38] Oranje A.P., Glazenburg E.J., Wolkerstorfer A., De Waard-Van Der Spek F.B. (2007). Practical issues on interpretation of scoring atopic dermatitis: The SCORAD index, objective SCORAD and the three-item severity score. Br. J. Dermatol..

[39] Mease P.J. (2011). Measures of psoriatic arthritis: Tender and Swollen Joint Assessment, Psoriasis Area and Severity Index (PASI), Nail Psoriasis Severity Index (NAPSI), Modified Nail Psoriasis Severity Index (mNAPSI), Mander/Newcastle Enthesitis Index (MEI), Leeds Enthesitis Index (LEI), Spondyloarthritis Research Consortium of Canada (SPARCC), Maastricht Ankylosing Spondylitis Enthesis Score (MASES), Leeds Dactylitis Index (LDI), Patient Global for Psoriatic Arthritis, Dermatology Life Quality Index (DLQI), Psoriatic Arthritis Quality of Life (PsAQOL), Functional Assessment of Chronic Illness Therapy-Fatigue (FACIT-F), Psoriatic Arthritis Response Criteria (PsARC), Psoriatic Arthritis Joint Activity Index (PsAJAI), Disease Activity in Psoriatic Arthritis (DAPSA), and Composite Psoriatic Disease Activity Index (CPDAI). Arthritis Care Res..

[40] Dassouli R., Elloudi S., Choukri S., Baybay H., Douhi Z., Mernissi F.Z. (2022). Dermoscopy of palmoplantar keratoderma: Development Dermoscopy of palmoplantar keratoderma: Development and analysis methods and analysis methods. Our Dermatol Online.

[41] Nuño-González A., De La Fuente E.G., Vicente-Martín F.J., López-Estebaranz J.L. (2012). Good Response of Hyperkeratotic Palmoplantar Psoriasis to Ustekinumab. Actas Dermosifiliogr..

[42] Kase R., Amemiya A., Okonogi R., Yamakawa H., Sugawara H., Tanaka Y.L., Komiyama M., Mori T. (2018). Examination of the Effect of Suitable Size of Shoes under the Second Metatarsal Head and Width of Shoes under the Fifth Metatarsal Head for the Prevention of Callus Formation in Healthy Young Women. Sensors.

[43] Sage R.A., Webster J.K., Fisher S.G. (2001). Outpatient Care and Morbidity Reduction in Diabetic Foot Ulcers Associated with Chronic Pressure Callus. J. Am. Podiatr. Med. Assoc..

[44] Hamada T., Tsuruta D., Fukuda S., Ishii N., Teye K., Numata S., Dainichi T., Karashima T., Ohata C., Furumura M. (2013). How do keratinizing disorders and blistering disorders overlap?. Exp. Dermatol..

